# The adaptive landscape of the miR482 gene family in tomato is rugged

**DOI:** 10.3389/fpls.2026.1884466

**Published:** 2026-10-01

**Authors:** Jessica Nicolai, Laura E. Rose

**Affiliations:** 1 Institute of Population Genetics, Heinrich-Heine University Duesseldorf, Duesseldorf, Germany; 2 Cluster of Excellence on Plant Sciences, Heinrich Heine University Duesseldorf, Duesseldorf, Germany; 3 DFG Research Consortium for Plant Ecological Genetics (TRR341), Duesseldorf, Germany

**Keywords:** adaptation, adaptive landscape, disease resistance, gene expression, microRNA, NBS-LRRs, resistosome

## Abstract

MicroRNA genes are important regulators of development and disease resistance in plants. Successful miR:target interactions rely on high sequence complementarity, usually permitting few to no mismatches. Exploring the concept of an adaptive landscape, we investigated how individual nucleotide changes in the miR482 gene family may alter the targeted repertoire of disease resistance gene (*R*-genes) in tomato. We predicted the target scores for all mutants accessible through individual point mutations *in silico* and compared them to the target scores of the original miR482 genes. Most mutations lead to a loss of targeting or lower likelihood of targeting. Only a few mutants showed improved target scores or gained new *R*-gene targets. Our results indicate that the miR482 genes may be optimized for targeting of their current *R*-gene repertoire. This suggests that the miR482 genes experience natural selection to maintain their current target repertoire and that the adaptive landscape is relatively peaked or rugged. This study has implications for understanding the adaptive process governing gene regulation in plants and the design of new miR:target pairs for generating novel, beneficial plant phenotypes.

## 1 Introduction

An adaptive landscape describes the relationship between genotype and fitness in a given environment (Wright, 1931). It can be visualized as a 3D landscape containing one or multiple peaks and valleys. Within the landscape, genotypes located in a valley have a lower relative fitness compared to genotypes located on a peak. If a genotype is located on a peak, mutations in that genotype lead to a reduction in fitness. Conversely, if a genotype is in a valley, mutations lead to an increase in fitness. A fitness landscape can also contain ridges linking peaks, accessible via multiple neutral mutations. The ridge may itself be an optimum where multiple genotypes share the same fitness or a ridge can represent a path between different peaks. Knowledge about the fitness landscape provides insights into the potential adaptability of a population and can reveal evolutionary constraints present in a population.

In this study, we evaluated the adaptive landscape of a microRNA family that targets disease resistance genes in plants. MicroRNAs (miRs) are small, non-coding RNAs (Lee et al., 1993) that regulate the expression of their target genes via post-transcriptional regulation (Reinhart et al., 2002). Gene regulation via miRs plays an important role in development (reviewed in Dong et al., 2022) and disease resistance (Zhang et al., 2019). The miR482 family negatively regulates the expression of resistance genes (*R*-genes) in tomato (Meyers et al., 2005; Shivaprasad et al., 2012; de Vries et al., 2015).

*R*-genes are key players in the plant immune system (Jones and Dangl, 2006). Their products, the R-proteins, act as receptors for effector molecules, secreted by pathogens to facilitate infection. Recognition of a pathogen effector activates an immune response (Glazebrook, 2005; Tsuda and Katagiri, 2010). The largest class of *R*-genes consists of a variable amino-terminal domain, a central conserved nucleotide binding site (NB-ARC), that contains the P-loop, and a carboxy-terminal leucine-rich repeat (LRR) region. The miR482 family binds to the conserved P-loop of the *R*-genes (Shivaprasad et al., 2012). For successful gene regulation, a high degree of reverse-complementarity between the miR and the target is necessary (Mallory and Bouché, 2008). However, some sequence variation is tolerated at the positions that bind to the 3^rd^ codon positions encoding the P-loop of the *R*-genes (Zhang et al., 2016). The miR482 gene family in tomato encodes six distinct mature 22 nt long miRs (**Figure 1A**) and targets 59 different tomato *R*-genes (**Figure 1B**). Some *R*-genes are targeted by multiple members of the miR482 family in tomato; for example, miR482a and miR482b, share about 2/3s of their targets (**Figure 1B**).

**Figure 1:**
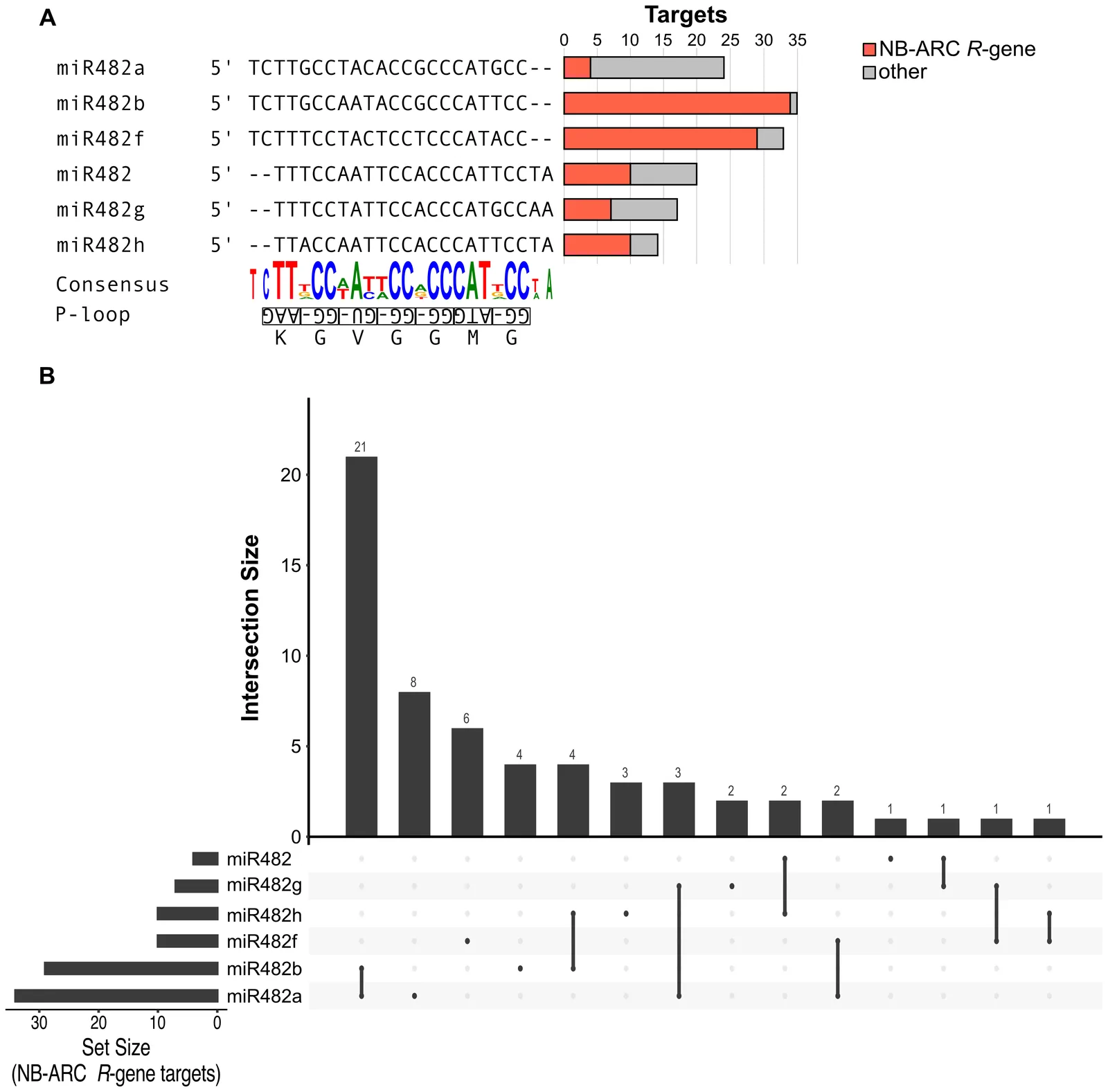
Overview of miR482 family targeting. **(A)** Aligned sequences of the six miR482 genes and their predicted NB-ARC R-gene (red) and other targets (grey). The consensus sequence was generated using WebLogo (Crooks et al., 2004). The alignment visualization is adapted from Canto-Pastor et al. (2019). **(B)** Upset plot showing shared and unique NB-ARC R-gene targets between the six miR482 genes.

Since NBS-LRR *R*-genes are some of the most rapidly evolving genes in plants (Rose et al., 2004, Rose et al., 2011), this raises the question: how do the members of the miR482 family maintain consistent targeting while their target genes are subjected to strong diversifying selection? Using an *in-silico* approach to predict how nucleotide substitutions in existing miR482 genes changed targeting, we explored the adaptive landscape of this small gene family. For each miR482 family member we inferred all 66 mutants that are accessible through a single point mutation. We compared the predicted targeting of the miR482 genes with their mutants to investigate how the mutations affect the target repertoire.

## 2 Materials and methods

### 2.1 Phylogenetic analysis

The NB-ARC domains from the tomato reference genome (Heinz 1706 SL4.0; Hosmani et al., 2019) were identified using NLRtracker (v1.0.3; Kourelis et al., 2021). The local multiple sequence alignment of the amino acid sequences of the NB-ARC regions from tomato was constructed using MAFFT (v7.273; Katoh and Standley, 2013). To identify the best fitting substitution model and infer the maximum likelihood tree, the Model Finder function of IQTREE (Nguyen et al., 2015; Kalyaanamoorthy et al., 2017) was used. The JTT+F+G4 model was selected, and 100 bootstrap replicates were evaluated.

### 2.2 MicroRNA: target prediction

The adaptive landscape of the miR482 family was constructed by enumerating the 66 1 mutant neighbors (1mut neighbors) of each miR482 gene (22 nt long) and inferring the predicted targets of these mutants. Potential targets in tomato were predicted with TargetFinder (v1.7; Fahlgren and Carrington, 2010). The coding sequences (CDS; ITAG4.0; Hosmani et al., 2019) of all known tomato genes were used as the target library. When multiple target sites were predicted in a single *R*-gene, the hit with the best score (i.e. the lowest score) was used. Lower target scores indicate a higher probability of targeting, while higher scores are less likely to result in targeting.

### 2.3 Differential targeting behavior

To analyze the changes in the targeting behavior of the miR sequences incurred by a single mutational step, we compared the target score of each *R*-gene target of a given miR482 gene with the target scores of its 66 1mut neighbors. The *R*-gene targets (in rows) were sorted based on their phylogenetic relationships (**Figure 2**; **Supplementary Table S1**) while the 1mut neighbors (in columns) were arranged according to the position of the mutation along the 22 positions of the original miR.

**Figure 2:**
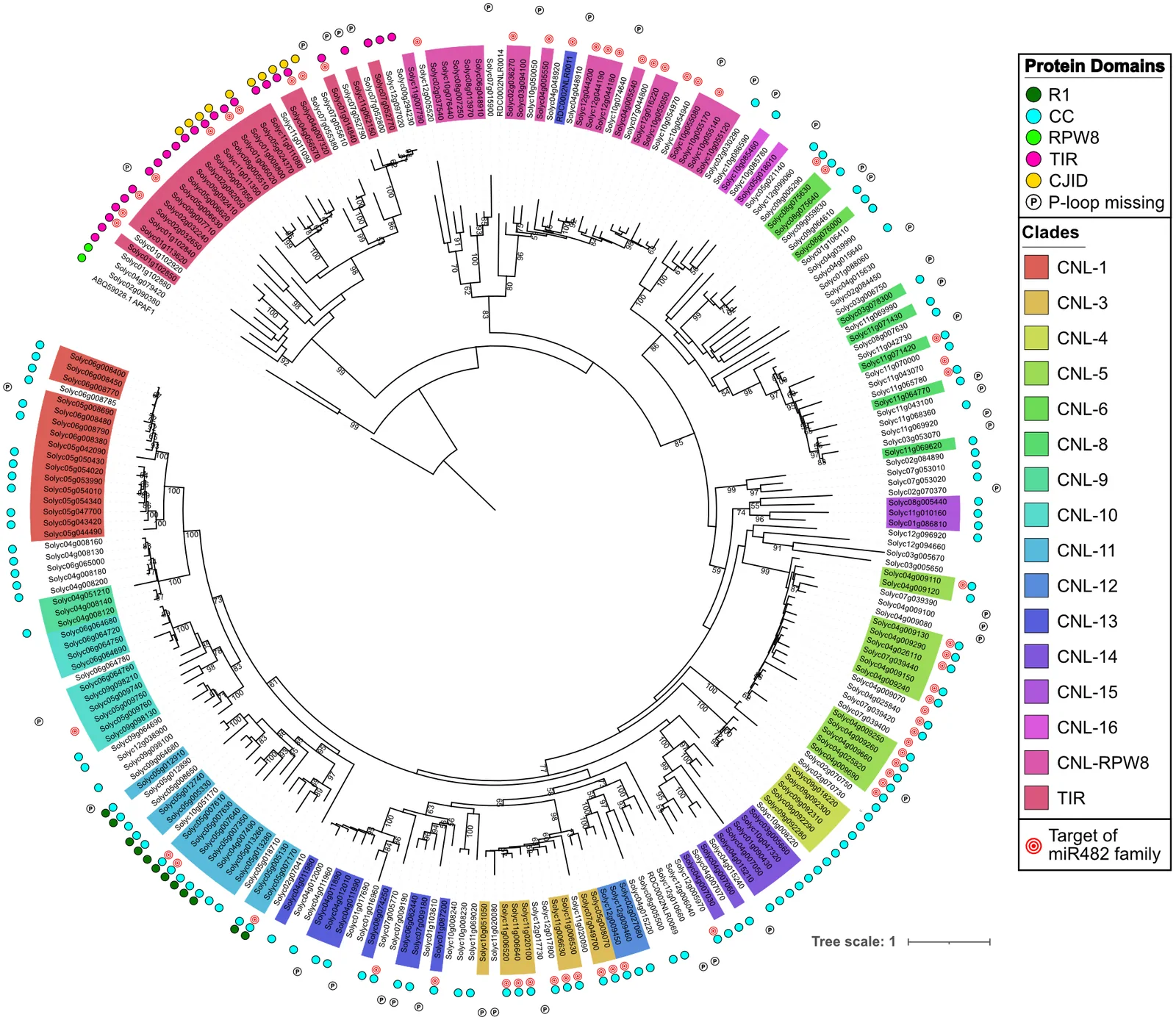
Phylogeny of tomato resistance genes. The NB-ARC domains of 242 tomato resistance genes were used together with the human APAF1 to infer a maximum likelihood tree using the JTT+F+G4 model. Clades identified by Andolfo et al., 2014 are highlighted accordingly. Identified protein domains and targeting by miR482 genes are indicated on the outer circles. Bootstrap values greater than 50 are shown.

### 2.4 Peakness

Using the approach proposed by Schweizer and Wagner (2021), we evaluated the peakness of the targeting landscape of each miR482 gene. Peakness is defined as the proportion of the neighbors with a lower probability of targeting compared to the original miR gene. Therefore, peakness can range from 0 to 1. A peakness value of 0 means all 66 1mut neighbors have a better or equal targeting score compared to the original miR, while a peakness value of 1 means that targeting is lower for all 1mut neighbors compared to the original miR. Therefore, higher peakness values indicate that most mutations lead to loss of targeting and that miR genes are situated on local peaks within the targeting landscape.

### 2.5 Statistical analysis

Kruskal-Wallis tests followed by Dunn’s pairwise comparisons (StatsCalculators Team, 2026) were performed to evaluate whether the 1mut neighbors of each miR gene differed in their predicted targeting behavior as well as to test for differences in peakness within the miR482 genes.

## 3 Results

### 3.1 The targeted *R*-genes form evolutionary clusters based on their additional N-terminal domains

To determine if the pattern of targeting by the miR482 genes was related to the evolutionary history of the targeted *R*-genes, we inferred a phylogenetic tree of the *R*-genes containing NB-ARC domains and labeled the genes targeted by miR482 genes (**Figure 2**). Due to the high degree of protein conservation of the NB-ARC domains, this domain has been shown to be reliable for inferring phylogenetic relationships of resistance genes (Meyers et al., 2002; Lee and Chae, 2020). A total of 242 NB-ARC domains from tomato were included. The human gene, *Apaf1.1*, which contains a NB-ARC domain, was used as an outgroup. Our phylogenetic analysis showed strong concordance with the phylogenetic tree reported by Andolfo et al. (2014). Both studies show a distinct separation of the TIR/TNL and CC/CNL resistance genes, although these domains were not used for inferring the phylogenetic tree in our study. Due to the high degree of similarity to the tree described in Andolfo et al. (2014) and for ease of comparison, we adopted their clade nomenclature here.

### 3.2 Most 1mut neighbors are predicted to lose targeting

To evaluate the targeting landscape of the miR482 gene family, the target scores of each miR482 gene were compared with the target scores of their 66 1mut neighbors. Collectively, genes in the miR482 family are predicted to target 59 of the 242 NB-ARC *R*-genes (24%; **Figures 1**, **2**). miR482a had the largest number of targets (34 *R*-gene targets) and miR482 had the smallest (4 *R*-gene targets). The results of the miR482f gene (**Figure 3**) were used for illustrative purposes. The results for the other miRs can be found in the supplemental data (**Supplementary Figures S1**–**S5**). For each of these miR482 genes, this study determined how targeting was predicted to be altered for each of the 66 1mut neighbors. For any given originally targeted *R*-gene, most 1mut neighbors were predicted to lose targeting after the introduction of a single point mutation (**Figure 3**; **Supplementary Figures S1**–**S5**). However, target loss was rarely offset with the gain of new targets.

**Figure 3:**
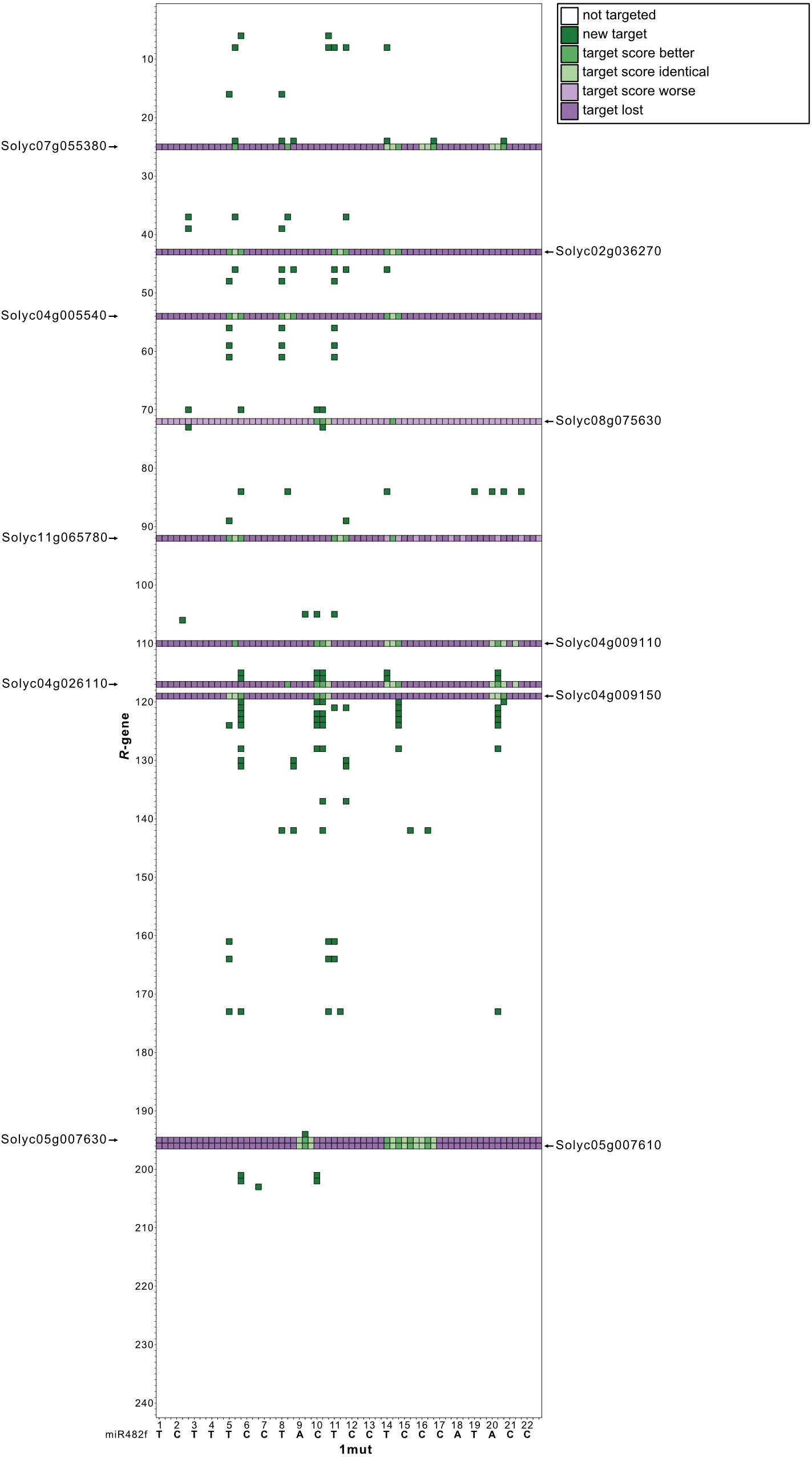
The target scores of the 66 1mut neighbors of miR482f of tomato R-genes. The 242 R-genes are in rows, sorted by phylogenetic position according to **Figure 2**. The gene names of the 10 original R-gene targets are labeled. The colors show the change in targeting behavior: target lost (dark purple), target score worse (light purple), target score identical (light green), target score better (medium green) and new target gained (dark green). The mutants are sorted alphabetically at each position.

Up to 59 out of the 66 possible 1mut neighbors (89%) were predicted to lose targeting of a particular *R*-gene, although the exact proportions varied across the different *R*-gene:miR combinations. Across all original targets and all miR482 genes, approximately three-quarters of 1mut neighbors were predicted to lose targeting (**Figures 3**, **4A**; **Supplementary Figures S1**–**S5**). The 1mut neighbors of miR482f had the highest proportion of target loss: for 9 out of the 10 *R*-gene targets, more than 75% of the 1mut neighbors were predicted to lose targeting (**Figures 3**, **4A**). For one specific *R*-gene target of miR482f (*Solyc08g075630*), none of the 1mut neighbors were predicted to completely lose targeting, but the target score was worse for most 1mut neighbors. In contrast, the 1mut neighbors of miR482h and miR482a had the lowest proportion of target loss (on average 55.5% and 55.7%).

**Figure 4:**
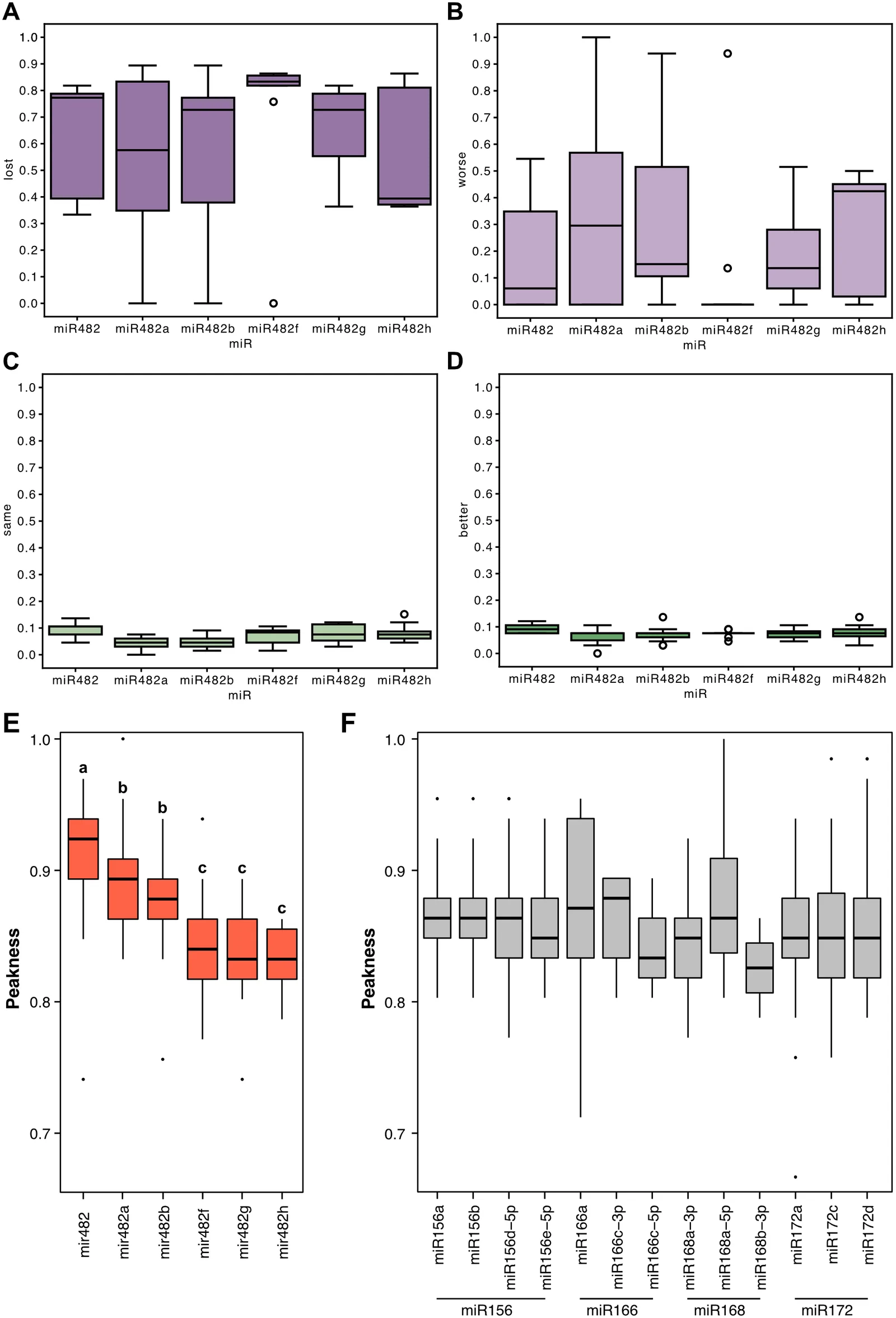
Boxplots of changes in predicted targeting and peakness of 1 mut neighbors of miR482 genes. **(A–D)** Displayed are the distributions of the 1mut neighbors that had **(A)** lost a target compared to the original miR or had a **(B)** worse, **(C)** same or **(D)** better target score compared to the original miR. **(E, F)** Peakness of the miR482 and reference families. The peakness was calculated across all predicted targets for **(E)** the miR482 genes and **(F)** the four reference miR families. The significant differences between the miR482 genes (p < 0.05) according to a Kruskal-Wallis test followed by Dunn’s pairwise comparison are indicated by letters a-c above the boxplots.

Single mutations can abolish the ability of a miR to target a gene or modify the likelihood of targeting. In cases in which targeting wasn’t abolished, most nucleotide changes were predicted to result in a reduced likelihood of targeting of original targets (i.e. higher target scores are associated with lower probability of targeting). Across all targets, between 11%-33% of the 1mut neighbors were predicted to have a lower probability of targeting a *R*-gene compared to the original miR482 gene (**Figures 3**, **4B**; **Supplementary Figures S1**–**S5**). miR482a had the highest proportion of 1mut neighbors showing a lower probability of targeting (33%), while miR482f had the lowest (11%; **Figure 4B**).

Although the majority of 1mut neighbors were predicted to lose targeting or have a lower likelihood of targeting relative to the original miR, a small proportion of 1mut neighbors were predicted to have the same or better target scores than the original miR482 gene. On average, 5%-9% of the 1mut neighbors had the same target score, while 7%-9% of the 1mut neighbors had a better target score (**Figures 4C, D**). miR482 had the highest proportion of 1mut neighbors with the same and better target score, while miR482a had the lowest proportion.

We performed Kruskal-Wallis tests followed by Dunn’s pairwise comparisons to determine if the proportions of the 1mut neighbors assigned to the four categories, based on their predicted change in targeting (lost, worse, same, or better), differed across miR482 genes. The proportions of the 1mut neighbors assigned to three categories (lost, worse and better) did not differ across the miR482 genes. However, the miR482 genes did differ in the proportions of their 1 mut neighbors for which targeting remained the same (Kruskal-Wallis test, p-value = 0.0007). Significant differences were found between two pairs: miR482a and miR482h (Dunn’s Test, p-value = 0.0275) and miR482b and miR482h (Dunn’s Test, p-value = 0.0491).

### 3.3 Potential expansion of the target repertoire through single nucleotide changes

This study examined if the 1mut neighbors were predicted to gain novel targets, i.e. were predicted to target *R*-genes that were not targeted by their original miR482 gene. Of the 242 *R*-genes included in our analysis (**Figure 2**), initially 183 were not predicted to be targeted by any of the original miR482 genes. Across all 396 possible 1mut neighbors, 121 of the 242 (50%) *R*-genes were gained as novel targets. Of those novel *R*-gene targets, 68 were predicted to be completely new *R*-gene targets, i.e. not targeted by any of the original miR482 genes. The remaining 53 novel targets were predicted to be targeted by an original miR482 gene and a 1mut neighbor of a different miR482 gene. Around 50% (190/396) of all possible 1mut neighbors gained at least one novel target. To fully capture the changes in the targeting landscape of 1mut neighbors, a more lenient upper cutoff of 11 was used for comparing the original miR482 genes and their 1mut neighbors (**Supplementary Figures S6**–**S11**). The target score distribution was only evaluated for *R*-genes for which the original miR482 gene had a target score of 5 or lower (i.e. likely true targets, **Supplementary Figure S9**, red dots). For most of the *R*-gene:miR combinations, the predicted target score of the original miR482 gene was in the top 25% of the distribution compared to the 1mut neighbors (**Supplementary Figure S9**). This corresponds to the heatmap analysis which indicated that most 1mut neighbors lost targeting or were less likely to target these *R*-genes (**Figures 3**, **4**).

### 3.4 The shape of the adaptive landscape

The shape of the adaptive landscape of miR targeting was evaluated by calculating the peakness for all targets (including non-*R*-genes) for the miR482 family as well as for members of the miR families: miR156, miR166, miR168 and miR172 (**Figures 4E, F**). These four miR families target developmental processes in plants and serve as a comparison for the miR482 family (Xian et al., 2014; Clepet et al., 2021; Lin et al., 2021; Barrera-Rojas et al., 2023). Peakness is defined as the proportion of the 1mut neighbors with a lower probability of targeting compared to the original miR gene. Therefore, peakness for a miR:target combination can range from 0 to 1. A peakness value of 0 corresponds to the situation in which all 66 1mut neighbors have a better or equal targeting score compared to the original miR, while a peakness value of 1 means that targeting is lower for all 1mut neighbors compared to the original miR. Higher peakness corresponds to a more rugged targeting landscape. Movement between peaks would likely require multiple mutations and be associated with an initial reduction of the number of targets. Loss of targeting means that the *R*-gene transcripts are not degraded and can be expressed as proteins. Most miR482 genes show a peakness of 0.8 or higher and fewer than 25% of miR:target combinations have a peakness below 0.8, with the lowest values around 0.74. One target (*Solyc04g009070*) of miR482a had a peakness of 1, meaning that all 1mut neighbors were less likely to target the gene. Likewise, the peakness of the four reference miR families was similar to the miR482 genes (**Figures 4E, F**). A Kruskal-Wallis test was used to test for significant differences in peakness within the miR482 family and within the reference miR families. Peakness differed significantly between the miR482 genes (H-Statistic= 61.1494; p-value = 0.0000). These genes fell into 3 groups: Group a) consisting of miR482, Group b) consisting of miR482a and miR482b and Group c) consisting of miR482f, miR482g and miR482h (**Figure 4E**).

### 3.5 Core *R*-genes and the miR482 targeting landscape

Disease resistance genes in tomatoes are typically expressed at low levels or only under specific environmental/experimental conditions (von Dahlen et al., 2023). However, a small subset of these genes shows moderate levels of expression across a range of environmental conditions and tissue types. In a previous study, we designated these genes as “core” *R*-genes (von Dahlen et al., 2023). Indeed, some of these core *R*-genes encode proteins present in an inactive form and only assemble into a well-defined resistosome upon activation (Ma et al., 2024). Due to their distinct patterns of constitutive gene expression relative to other *R*-genes and negative post-translational regulation, we reasoned that this subset of genes would not be targeted by the members of the miR482 family. We predicted the targeting of the 14 core *R*-genes present in our dataset by the miR482 genes and their 1 mut neighbors. None of the original miR482 genes target these core *R*-genes. However, a small number of 1mut neighbors were predicted to gain these core *R*-genes as novel targets (*Solyc01g090430*, *Solyc01g102840*, and *Solyc04g079420*). In all three cases, the novel targets were gained each time by 1mut neighbors of a single miR482 gene (1x miR482, 2x miR482b). Assuming that the constitutive expression of core *R*-genes is evolutionarily beneficial, then a gain of targeting by novel miR variants (thereby inducing gene silencing) may represent a deleterious mutation. Therefore, changes in the *R*-gene targeting repertoire must be balanced against potential deleterious consequences of silencing essential genes at the wrong time or place. This may also be one clue to the ruggedness of the miR targeting landscape – to prevent rapid shifts in the targeting repertoire through only few mutational steps.

## 4 Discussion

miRs are important regulators of development and disease resistance in plants (Zhang et al., 2019; Dong et al., 2022). This study focuses on the miR482 family in tomato which negatively regulates the expression of NB-ARC *R*-genes in tomato (Meyers et al., 2005; Shivaprasad et al., 2012; de Vries et al., 2015, de Vries et al., 2018). Although previous studies have focused on the evolutionary history and target specificity of the miR482 family (de Vries et al., 2015, de Vries et al., 2018) or miR gene expression and impact on disease resistance (Canto-Pastor et al., 2019), the adaptive landscape of the miR482 family has not been considered. The adaptive landscape provides insights into how easily nucleotide changes in miR genes can modulate the repertoire of the expressed *R*-genes. Since one irreversible outcome of *R*-gene activation can be cell death, *R*-genes are tightly regulated in plants. Therefore, strong purifying selection likely operates to ensure the proper expression of these genes. Furthermore, since the miR:target interaction is highly sequence specific, usually permitting only few to no mismatches (Schwab et al., 2006), we anticipated that single nucleotide changes could potentially impact which *R*-genes are repressed or expressed. In this study, we observed that most mutations were predicted to lead to a loss of targeting or lower likelihood of targeting. This implies that these miRs are optimized for targeting their existing set of *R*-genes and suggests a relatively peaked or rugged evolutionary landscape. Furthermore, predicted target loss was not always matched by a gain of new targets. This asymmetry in target loss versus gain is significant, because it implies that single nucleotide changes may easily allow previously repressed genes to become activated, but these same miR variants are less likely to target (and repress) new genes. This implies that target loss is mutationally permissive (which could lead to promiscuous *R*-gene expression), while target gain (*R*-gene repression) is less permissive.

The mutational asymmetry in target loss versus gain is intriguing because it is likely that target gain by miR482 genes could be deleterious depending on which NBS-LRRs are targeted, since these changes could potentially repress important genes. The class of NBS-LRRs in plants is diverse, with some serving as specific pathogen sensors and others acting downstream of pathogen perception (Toghani et al., 2025). Some of these NBS-LRRs acting downstream encode proteins that assemble into multimeric resistosome structures upon protein activation and form calcium channels at the plasma membrane (Wang et al., 2023). The expression pattern of these downstream (or helper/core) NBS-LRRs is distinct from the other NBS-LRRs. Helper/core NBS-LRRs are often expressed across many tissues and experimental conditions (von Dahlen et al., 2023). Targeting of these helper/core *R*-genes by the miR482 genes would likely be deleterious. Indeed, none of the NBS-LRRs classified as core genes are currently targeted by miR482 genes and few of the 1mut neighbors gain this set as targets. Furthermore, some of these core genes and their encoded proteins have other forms of negative regulation. One recently discovered example in tomato is the NRC2 protein, an NBS-LRR which forms dimers in the inactive state (Ma et al., 2024). Therefore, we predict that most helper genes should not be targeted by the miR482 genes and are likely to have an independent form of negative regulation that may operate at the protein level, to ensure that resistance is expressed precisely when and where it is needed.

In addition to the mutational asymmetry in target gain and loss, the miR families showed a high degree of peakness, indicating that the miR genes may experience natural selection to maintain their targets. Few 1mut neighbors show improved target binding. If, however, the loss of targeting or the gain of new targets becomes advantageous, gene duplication, followed by divergence, would be one possible route for an organism to retain the current targeting repertoire and simultaneously acquire new targets. Comparative genomic studies indicate that these six miR482 genes are shared across the clade of cultivated and wild tomatoes and do not show rapid turnover across these species (de Vries et al., 2015). This implies that the evolutionary landscape is rugged, but also stable, at least within the tomato clade.

Many 1mut neighbors of the miR482 genes are predicted to lose targeting of multiple NBS-LRRs. These mutants may behave as miR knock-outs or silenced miRs. Several studies in tomato report negative fitness consequences when miRs are silenced, implying the action of strong purifying selection against the loss of targeting. For example, silencing of miR319 leads to smaller and simpler leaves as well as smaller plants overall (Sha et al., 2014). Silencing of miR167a leads to delayed fruit ripening (Duan et al., 2024). The pattern is different for miRs targeting disease resistance. Hong et al. (2021) showed that silencing of miR482a in tomato increased disease resistance against *Phytophthora infestans*. Simultaneous silencing of two miRs, miR482a and miR482b, increased disease resistance even further. Likewise, in rice, silencing of miR168 increased the resistance against the rice blast fungus *Magnaporthe oryzae* and increased yield (Liu et al., 2019). In these two studies, silencing of miRs was evaluated in the context of two specific pathogens, respectively. To what degree these fitness differences translate more broadly to a wide range of experimental conditions is unclear. Nevertheless, these studies do indicate that under certain circumstances, loss of gene silencing by specific miRs can be beneficial.

Since our analyses indicate that loss of targeting for the miR482 genes requires relatively few genetic changes and according to some experimental studies, these changes may be considered beneficial, it is a puzzle why the miR482 family shows limited sequence diversity within and between wild tomato species (de Vries et al., 2015). This implies that natural selection conserves the miR sequences, consistent with the slow rate of evolution and maintenance of miR482 gene family size across the tomato clade (de Vries et al., 2015).

Our study of the adaptive landscape of the miR482 family implies a fairly rugged, but stable landscape where target loss is permitted, while target gain is less likely. This study provides clues to why the targeting landscape is so sparse and why many NBS-LRRs show independent mechanisms of regulation. Furthermore, given the importance of precise gene regulation for disease resistance and yield, we anticipate that similar methods adapting the concept of an adaptive landscape can be used to predict and design novel miR:target pairs important for generating novel, beneficial plant phenotypes.

## Data Availability

Publicly available datasets were analyzed in this study. This data can be found here: https://solgenomics.net/ftp/tomato_genome/annotation/ITAG4.0_release/.
