# Supplementary material for: The adaptive landscape of the miR482 gene family in tomato is rugged
Source: Front Plant Sci. 2026 Oct 1;17:1884466. doi: 10.3389/fpls.2026.1884466 (PMC13668569; doi:10.3389/fpls.2026.1884466)
Supplement: Supplementary file 1 [file DataSheet1.pdf]

Supplementary Material

1 Supplementary Data

2 Supplementary Figures and Tables

2.1 Supplementary Figures

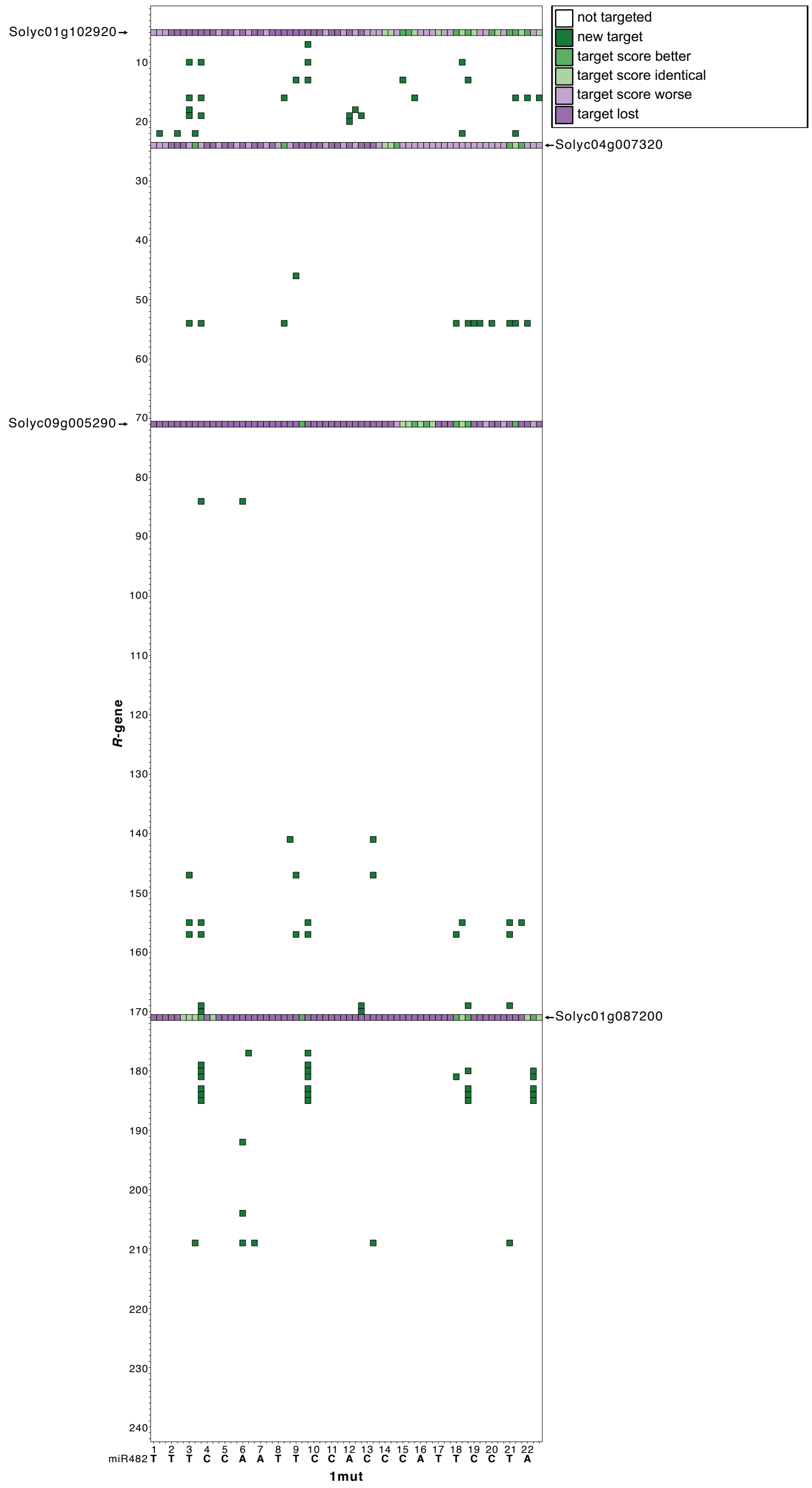

Figure S1. The target scores of the 66 1mut neighbors of miR482 of tomato R-genes. The colors show the change in targeting behavior: target lost (dark purple), target score worse (light purple), target score identical (light green), target score better (medium green) and new target gained (dark green). The mutants are sorted alphabetically at each position.

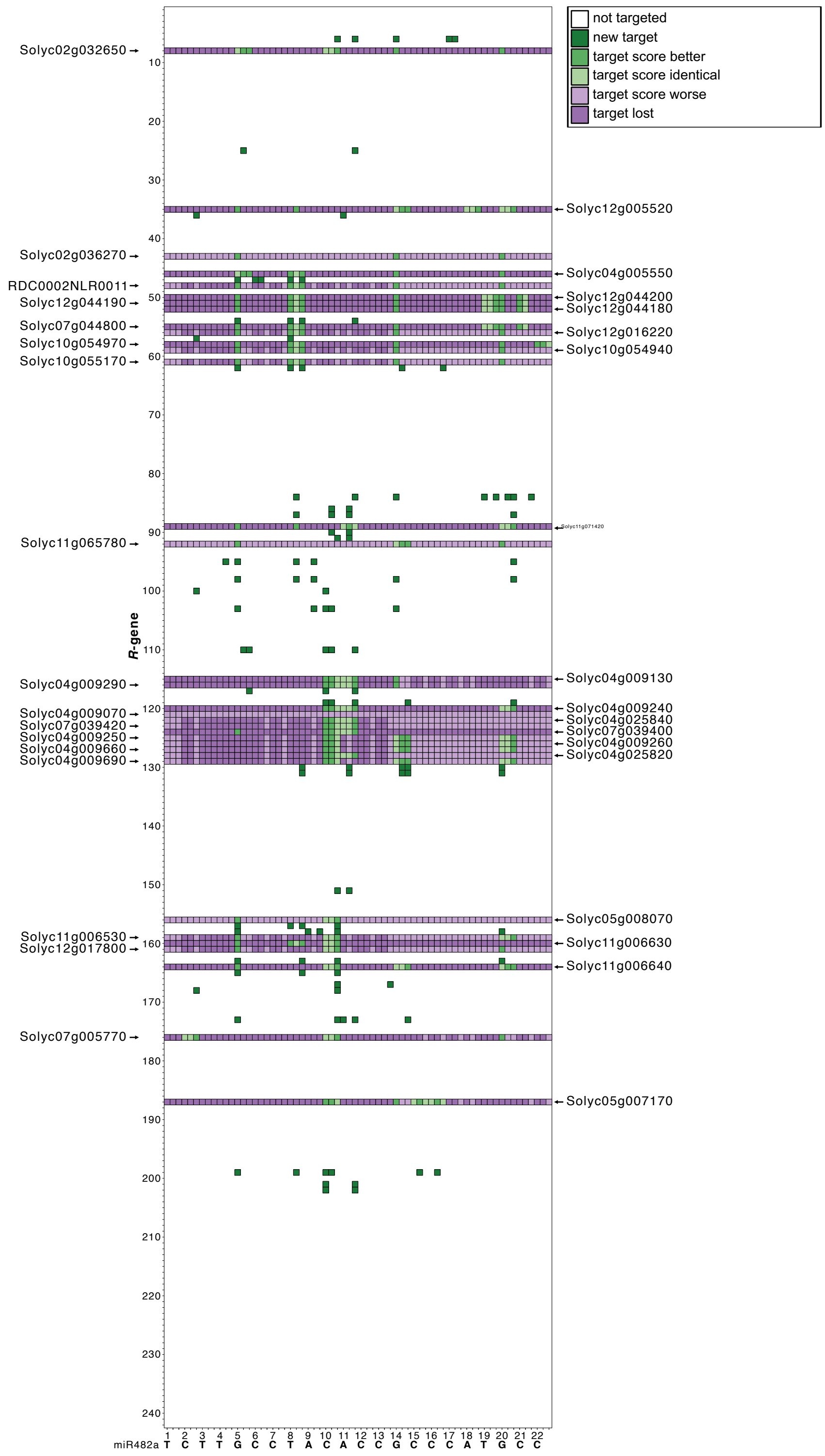

Figure S2. The target scores of the 66 1mut neighbors of miR482a of tomato R-genes. The colors show the change in targeting behavior: target lost (dark purple), target score worse (light purple), target score identical (light green), target score better (medium green) and new target gained (dark green). The mutants are sorted alphabetically at each position.

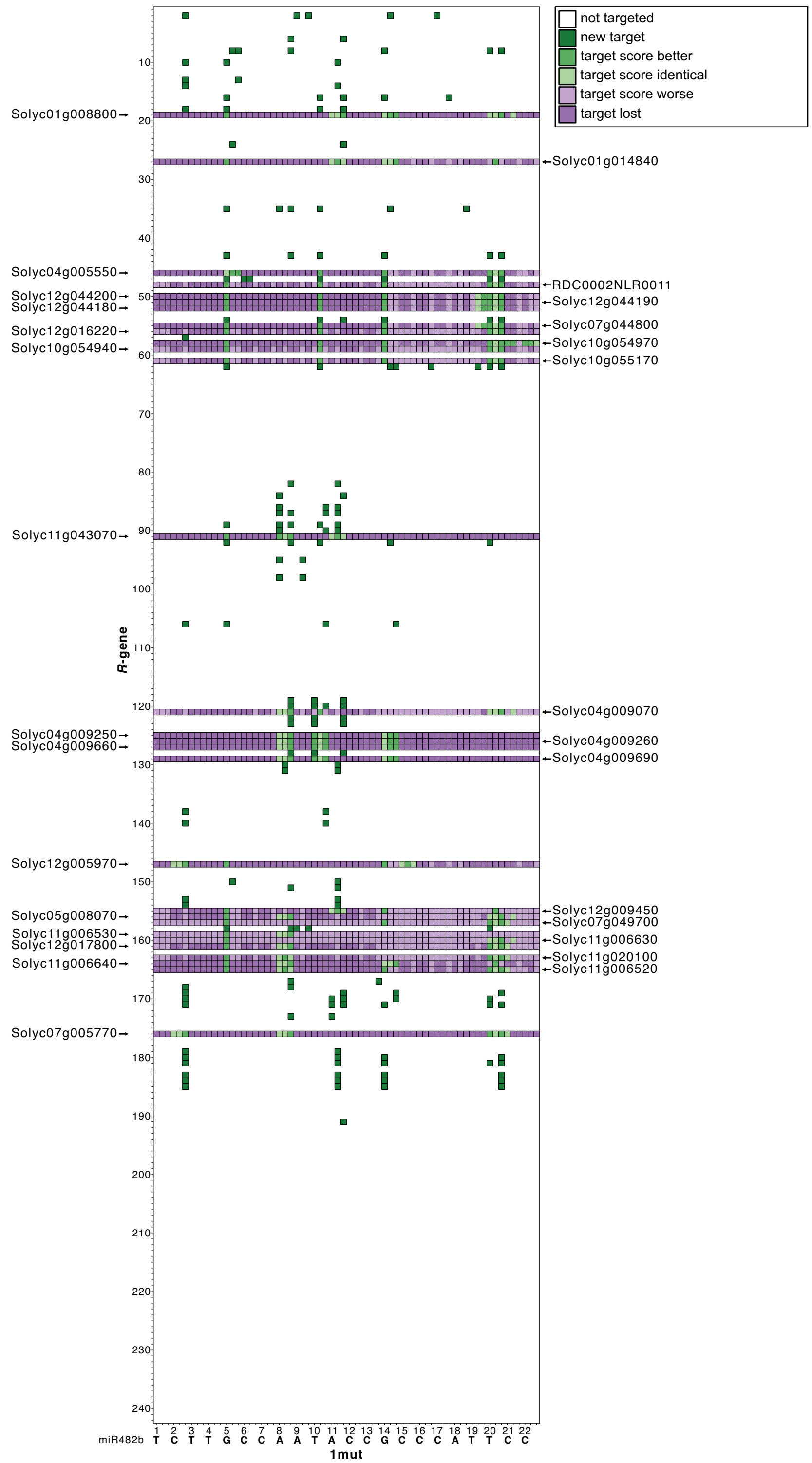

Figure S3. The target scores of the 66 1mut neighbors of miR482b of tomato R-genes. The colors show the change in targeting behavior: target lost (dark purple), target score worse (light purple), target score identical (light green), target score better (medium green) and new target gained (dark green). The mutants are sorted alphabetically at each position.

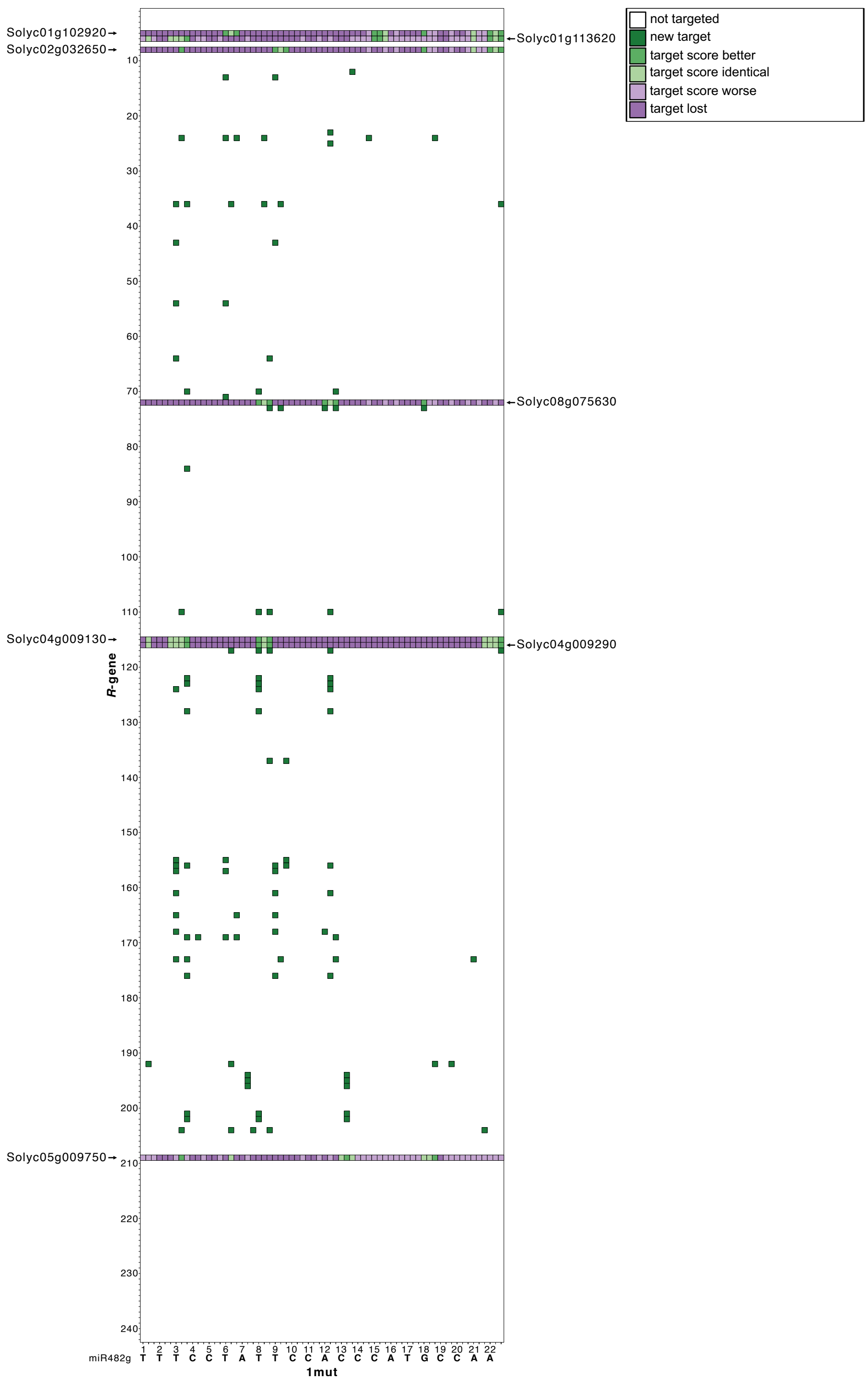

Figure S4. The target scores of the 66 1mut neighbors of miR482g of tomato R-genes. The colors show the change in targeting behavior: target lost (dark purple), target score worse (light purple), target score identical (light green), target score better (medium green) and new target gained (dark green). The mutants are sorted alphabetically at each position.

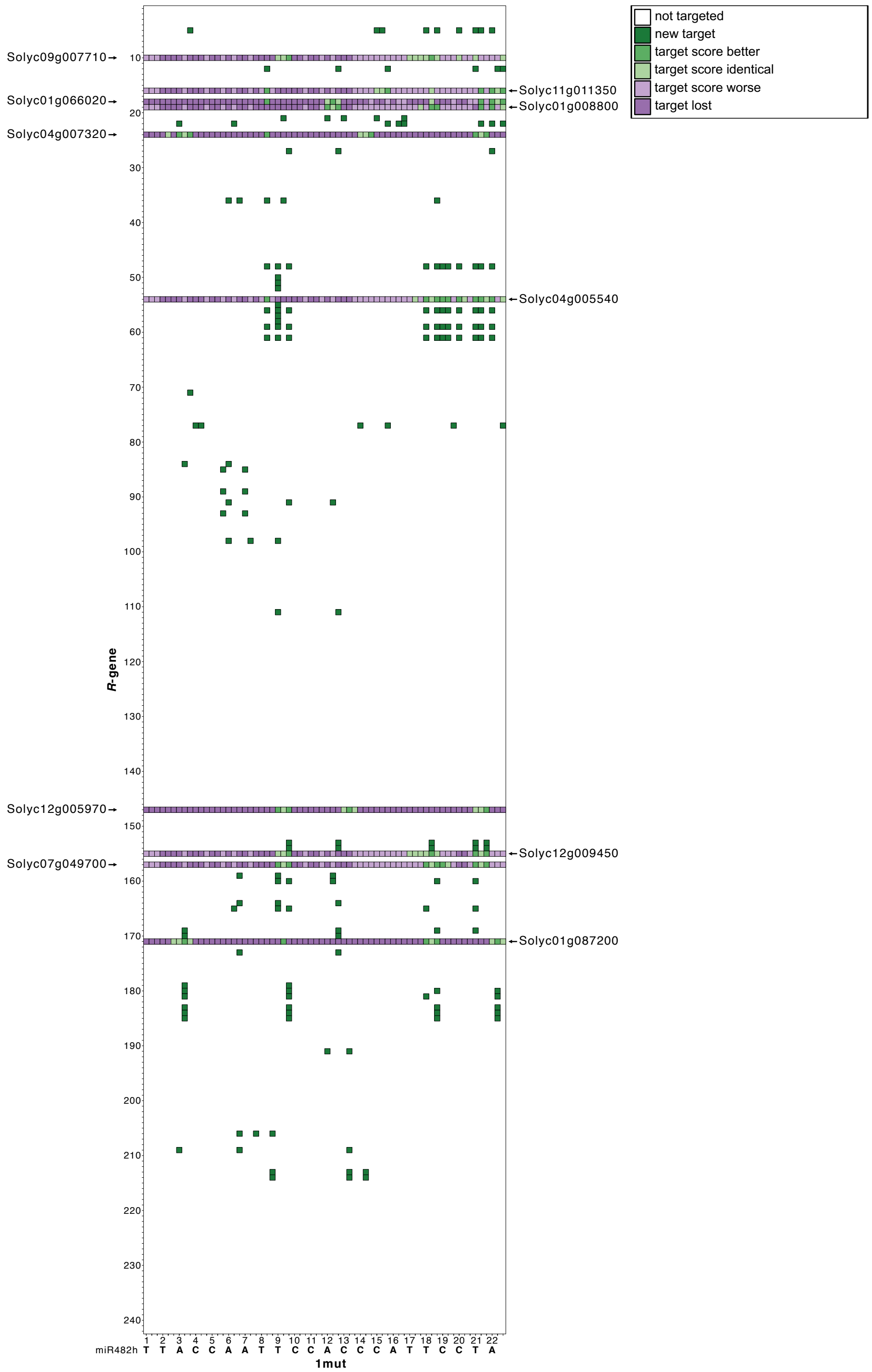

Figure S5. The target scores of the 66 1mut neighbors of miR482h of tomato R-genes. The colors show the change in targeting behavior: target lost (dark purple), target score worse (light purple), target score identical (light green), target score better (medium green) and new target gained (dark green). The mutants are sorted alphabetically at each position.

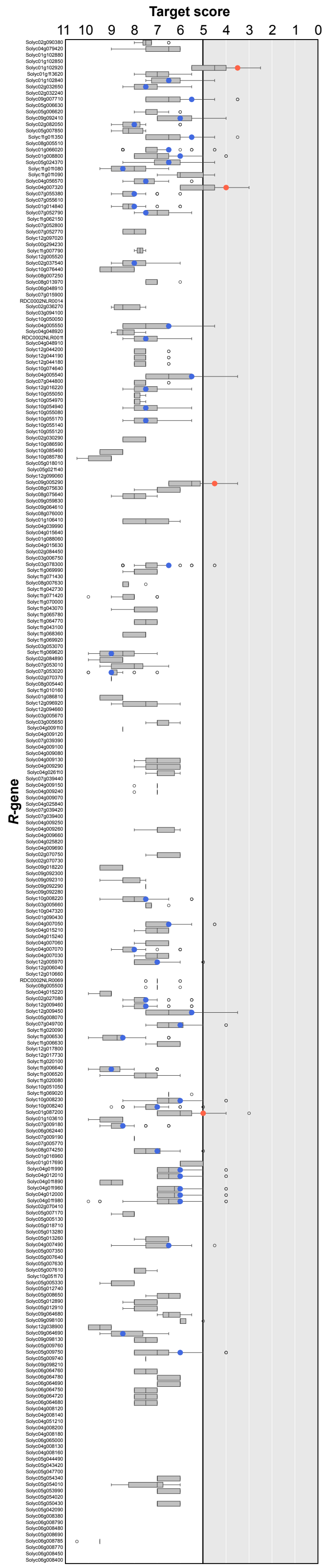

Figure S6. Boxplot distribution of the target scores of the R-gene targets of the 1mut neighbors of miR482. The predicted target score of the original miR482f gene is shown as a colored dot, divided in <= 5 (red) and >5 (blue).

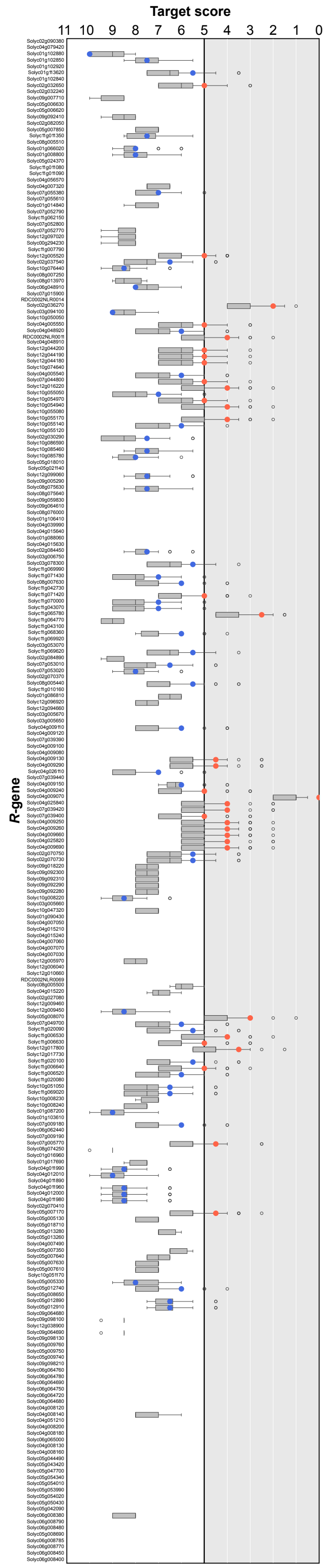

Figure S7. Boxplot distribution of the target scores of the R-gene targets of the 1mut neighbors of miR482a. The predicted target score of the original miR482f gene is shown as a colored dot, divided in <= 5 (red) and >5 (blue).

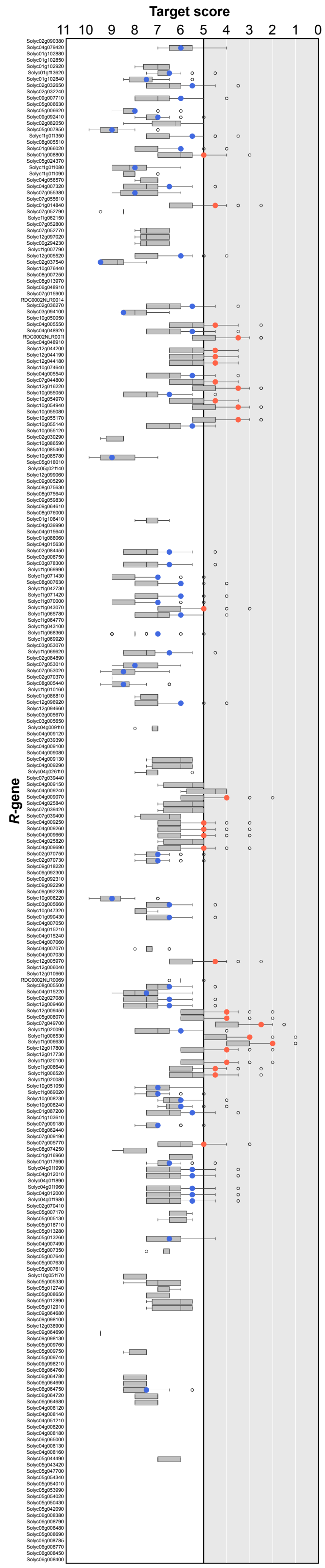

Figure S8. Boxplot distribution of the target scores of the R-gene targets of the 1mut neighbors of miR482b. The predicted target score of the original miR482f gene is shown as a colored dot, divided in <= 5 (red) and >5 (blue).

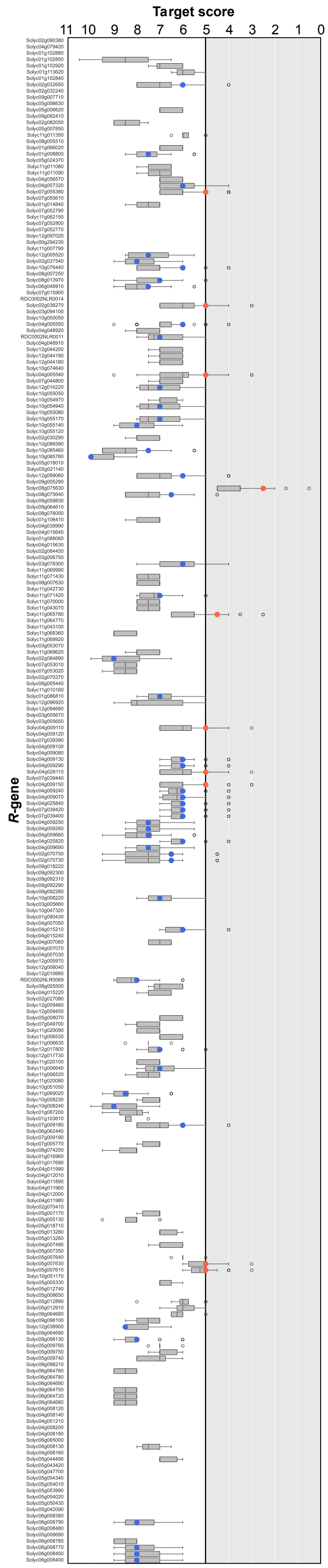

Figure S9. Boxplot distribution of the target scores of the R-gene targets of the 1mut neighbors of miR482f. The predicted target score of the original miR482f gene is shown as a colored dot, divided in <= 5 (red) and >5 (blue).

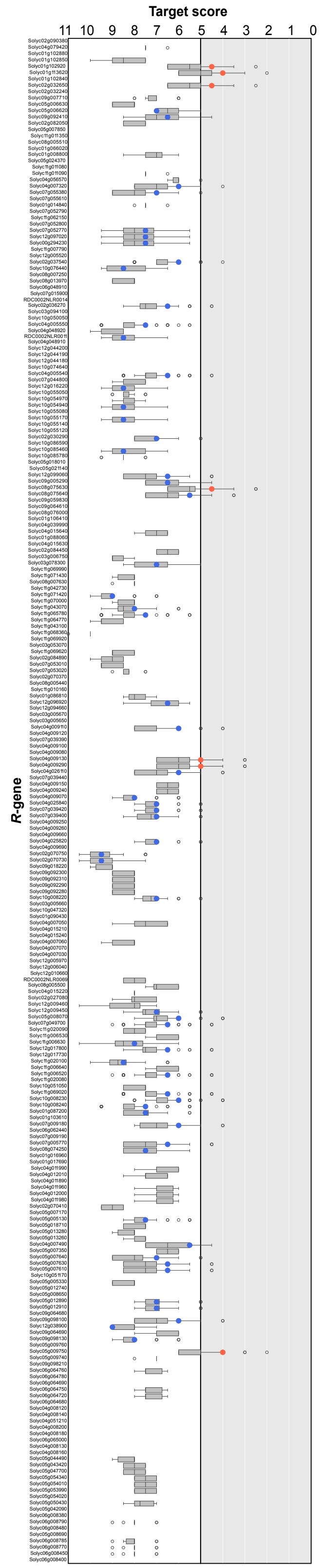

Figure S10. Boxplot distribution of the target scores of the R-gene targets of the 1mut neighbors of miR482g. The predicted target score of the original miR482f gene is shown as a colored dot, divided in <= 5 (red) and >5 (blue).

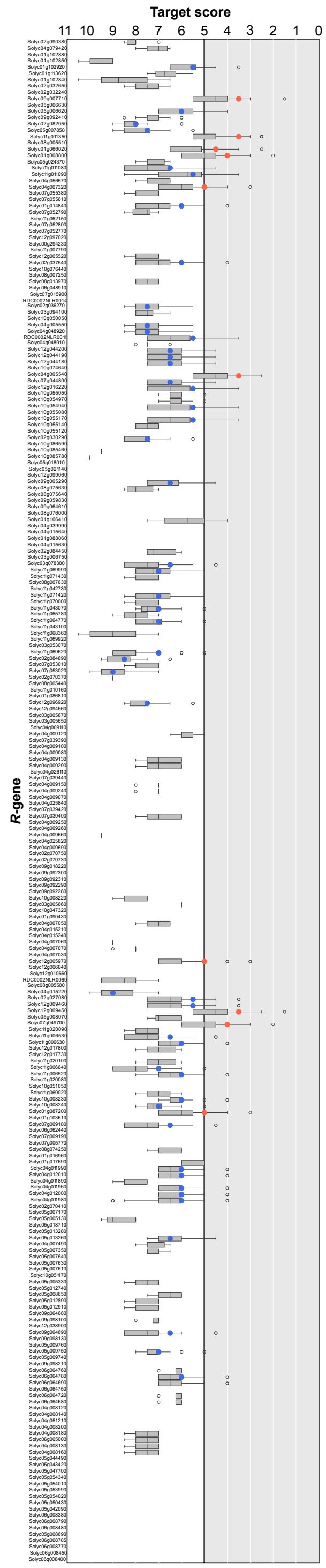

Figure S11. Boxplot distribution of the target scores of the R-gene targets of the 1mut neighbors of miR482h. The predicted target score of the original miR482f gene is shown as a colored dot, divided in <= 5 (red) and >5 (blue).

2.2 Supplementary Tables

Table S1 R-gene targets sorted based on their phylogenetic relationships. The table was submitted separately in 'Supplemental Table S1.xlsx'
